# Exploring age-related inhibitory deficits in auditory attention: Evidence from attention switching

**DOI:** 10.1007/s00426-026-02306-5

**Published:** 2026-05-11

**Authors:** Luigi Falanga, Thomas Deutsch, Janina Fels, Klaus Willmes, Denise N. Stephan, Iring Koch

**Affiliations:** 1 Institute of Psychology, RWTH Aachen University, Aachen, Germany; 2 Institute for Hearing Technology and Acoustics, RWTH Aachen University, Aachen, Germany; 3 Department of Neurology, RWTH Aachen University, Aachen, Germany

## Abstract

In multi-talker environments, inhibitory deficits may increase interference from auditory distractors and impair selective listening. To investigate whether age-related inhibitory deficits contribute to reduced control of auditory attention, we tested 45 younger and 45 older adults in a cued selective listening task. In each trial, a visual cue guided attention toward one of three sound sources (A, B, or C). Unlike previous studies that included immediate (n-1) location repetitions, the present task required switching on every trial. Participants categorised the magnitude of a spoken digit presented at the cued location while ignoring a simultaneous distractor at a different location. To assess target-distractor segregation, we measured performance costs when distractors elicited incongruent responses compared to congruent responses (congruency effect). We manipulated the n-2 location sequence to test whether returning to a previously attended location results in a cost (inhibition) or a benefit (facilitation). Results showed no age-related differences in the congruency effect, suggesting comparable target-distractor segregation across age groups. When participants switched attention to a previously attended location (ABA) relative to a new one (CBA), an n-2 location repetition benefit was observed, suggesting similar facilitation across groups. Older adults, in addition to general slowing, showed an n-2 location repetition benefit in error rates. Overall, these findings provide no evidence for an age-related inhibitory deficit during selective listening, even when accounting for age-related hearing loss. Instead, they suggest that auditory attention remains largely preserved in healthy aging, with older adults effectively resisting distractor interference and flexibly orienting attention across different sound sources.

**Supplementary Information:**

The online version contains supplementary material available at 10.1007/s00426-026-02306-5.

## Introduction

Under challenging listening conditions, effective control of auditory attention is essential for selecting the relevant acoustic signal while resisting interference from competing information (Bronkhorst, 2015; see Ihlefeld & Shinn-Cunningham, 2008, for a review). Previous studies have shown that listening difficulties in older adults cannot be fully explained by age-related sensory decline or by the general slowing of information-processing speed (Salthouse, 1996; Shende et al., 2021). However, the cognitive processes that support the control of auditory attention and how they decline with age are still poorly understood (Murphy et al., 1999, 2006; Shende & Mudar, 2023).

Theoretical frameworks of cognitive aging (Anderson & Craik, 2017; Hasher & Zacks, 1988) suggest that age-related deficits across a variety of cognitive tasks may arise from a decline in inhibitory processes (Forte et al., 2024; McDowd, 1997; see Rey-Mermet & Gade, 2018, for a review and meta-analysis). Inhibitory processes are fundamental both for resistance to distractor interference (Hasher et al., 1991; Mackie et al., 2013; Miyake et al., 2000) and for supporting flexible goal-directed behaviour in response to changes in the environment (Kiesel et al., 2010; Koch et al., 2010). Within the auditory domain, inhibitory control may be required both during target-distractor segregation (Kane et al., 1994; Nolden et al., 2019) and when switching attention to a newly relevant speaker or location (Koch et al., 2011).

The present study investigates age-related differences in the control of auditory attention and evaluates whether deficits in inhibitory processes contribute to these differences. To this end, we introduce a novel cued selective listening task to assess whether age-related differences in the congruency effect and the n-2 location repetition effect would emerge. While the former is a well-known behavioural marker reflecting target-distractor segregation (Kiesel et al., 2010), the latter is a proxy of the n-2 repetition costs often observed in task-switching studies and is here adapted to assess the potential contribution of inhibitory control processes during auditory attention switching (Koch et al., 2010; Mayr, 2001).

Early studies on selective listening employed dichotic-listening paradigms to investigate how auditory attention supports target processing and the suppression of irrelevant speech (Bronkhorst, 2015; Cherry, 1953). Since the well-known “cocktail party effect”, research on auditory attention has increasingly focused on the role of cognitive control processes in resolving distractor interference and in supporting attention switching between different sources of acoustic inputs (Getzmann et al., 2023; Nolden et al., 2019). To capture the role of cognitive control processes in auditory attention, Koch and colleagues (2011) developed a cued selective listening paradigm that integrated elements of task-switching into a dichotic-listening task.

On each trial, a visual cue indicated the relevant speaker’s gender (female vs. male voice). Following the cue, two different digits (from 1 to 9, excluding 5) were presented simultaneously, one in each ear, spoken by speakers of different genders (always one male and one female). Based on the cued gender, participants selected the digit spoken by the relevant speaker, and categorised it as smaller or larger than 5, while ignoring the digit spoken by the competing speaker in the (non-cued) gender. Across trials, the cued gender could either repeat or switch. According to the task-switching framework, the reduced performance observed on switch trials compared to repeat trials (i.e., switch costs) provides a behavioural marker of how flexibly participants reconfigure their attentional filter or attention set (Lawo et al., 2014; Monsell et al., 2019; Nolden et al., 2019).

This paradigm also allows for the investigation of participants’ ability to segregate target and distractor information. On each trial, the two spoken digits could be congruent (both digits smaller or both greater than 5), prompting the same response, or incongruent (one digit smaller and one larger than 5), requiring conflicting responses. Participants consistently show slower and more error-prone responses in incongruent trials compared to congruent ones (i.e., congruency effect), reflecting failures in distractor suppression, which then interferes with target processing and response selection (see Kiesel et al., 2010, for a review). Therefore, while the congruency effect may serve as a behavioural marker of target-distractor segregation in this task, the mechanisms underlying this effect remain not fully understood. Yet, inhibitory processes have been proposed as one possible explanation (Koch et al., 2011).

Subsequent studies have extended this work to examine age-related differences in auditory switch costs and congruency effect (Falanga et al. 2026; Lawo and Koch 2014b; Oberem et al. 2017). Using Koch et al.’s (2011) listening taskLawo and Koch (2014b) observed general slowing in the group of older listeners relative to the younger one. Such slowing is well established in the aging literature and is thought to reflect reduced sensory processing speed and delayed response selection and execution (Pichora-Fuller, 2003; Salthouse, 1996). However, comparable accuracy between younger and older adults, together with the absence of specific age-related differences in auditory switch costs and congruency effect, suggested no evidence for cognitive decline in the control of auditory attention (Lawo and Koch 2014b). Importantly, the listening setup used in this study provided a relatively simple auditory environment that, unlike a real-life auditory environment, may have reduced the cross-talk or energetic masking between competing signals at the perceptual level (Bronkhorst, 2015). In dichotic listening, with each speech stream delivered separately to a different ear, listeners can use lateralised cues to support spatial release from masking, facilitating target-distractor segregation at the peripheral level (Calcus et al., 2020). MoreoverLawo and Koch (2014b) did not report an assessment of participants’ hearing capacity, which may be relevant when interpreting age-related differences in selective listening performance.

Building on these methodological considerations, Oberem et al. (2017) extended the investigation by accounting for individual differences in sensory hearing capacity and implementing a more complex multi-talker listening environment. In their study, competing speech streams were presented binaurally from two out of eight spatially separated loudspeakers surrounding the listener. Compared to dichotic listening, binaural presentation allows each speech stream to reach both ears, increasing energetic masking between peripheral competing signals and increasing the cost of successful target-distractor segregation (Bronkhorst, 2015; Calcus et al., 2020). While older adults showed general slowing and reduced accuracy in overall listening performance, switch costs were similar across age groups. In this study, the congruency effect was modulated by the spatial position of the sound sources, with poorer target-distractor segregation for sources located in the median plane (front-back positions relative to the listener).

Importantly, older adults exhibited a larger overall congruency effect than younger adults. Yet, this age-related difference disappeared once hearing thresholds were included in the analysis, suggesting that sensory decline partially explained age-related differences in segregating competing inputs. Together, these results suggested that auditory attention is largely preserved in older adults, although hearing loss partially contributes to selective listening difficulties (Caso et al., 2024).

A more recent study by Falanga et al. (2026) investigated the relative contributions of sensory and cognitive factors during selective listening using a binaural setup in which, compared to Oberem et al. (2017), the number of possible loudspeakers was reduced to two (left and right relative to the participant’s head). This design reduced spatial complexity while preserving the energetic masking between peripheral competing signals inherent to binaural presentation (Calcus et al., 2020). Importantly, the study included larger samples of younger and older adults and controlled for individual differences in sensory hearing capacity by assessing pure-tone audiometry thresholds. This assessment allowed the inclusion of participants who, according to the World Health Organisation (WHO; Chadha et al., 2021), presented non-pathological hearing loss. Under these conditions, increased switch costs were found in both reaction time and error rate for older adults. These novel findings suggest that age-related differences in auditory attention switching may become detectable when sufficient statistical power is reached, and individual variability in sensory hearing capacity is controlled for. Importantly, because hearing thresholds were included as a covariate in the analyses, this pattern points to an age-related decline in the cognitive control processes required to reconfigure attention sets and to switch auditory attention that cannot be explained by sensory decline alone. Furthermore, age-related impairments in target-distractor segregation emerged specifically in switch trials, suggesting that for older listeners, residual activation from the previously relevant attention set biased the processing of incoming stimuli in the following trial, generating failures in target-distractor segregation (Falanga et al., 2026).

Although this pattern suggests greater difficulties in older adults when switching between different sound sources (Giller & Beste, 2019; Kramer & Kray, 2006; Schils et al., 2025; see Wasylyshyn et al., 2011, for a meta-analysis), the mechanisms underlying auditory attention switch costs, and thus the processes driving these age-related differences, remain uncertain. Specifically, auditory switch costs may result either from residual activation of the previously relevant set or from persisting inhibition of the previously ignored set (Koch et al., 2010; Monsell, 2003). Consequently, while age-related differences in switch costs and target-distractor segregation are consistent with the idea of reduced attentional control in older adults, they do not, by themselves, allow for a clear conclusion that such differences stem from an inhibitory deficit. These findings, therefore, provide initial evidence for an age-related decline in the cognitive control of auditory attention, while highlighting the need for paradigms designed to disentangle the underlying mechanisms (Koch et al., 2010).

Building on previous task-switching studies investigating age-related inhibitory deficits (Lawo et al., 2012; Mayr, 2001; Schuch, 2016), here, we asked whether reduced efficiency in inhibitory control underlies the increased auditory switch costs and congruency effect observed in older adults (Falanga et al., 2026). To address this question, we extended the cued selective listening paradigm implemented by Falanga et al. (2026) from two to three possible sound sources and adapted a well-established marker of inhibition from the task-switching literature: the n-2 repetition cost.

In task-switching studies (Lawo et al., 2012; Mayr & Keele, 2000; Schuch, 2016; see Koch et al., 2010) where participants alternate among three tasks (e.g., A, B, and C), performance is typically impaired when performing a recently abandoned task (ABA trials) compared to switching to a new task (CBA trials). These n-2 repetition costs are interpreted as evidence for inhibitory control: when a task set is abandoned, it is inhibited to reduce interference when switching to a new task, and this inhibition may persist, impairing performance when returning to the previously inhibited task (see Koch et al., 2010, for a review). By transferring this logic to auditory attention switching, our novel approach allowed us to investigate the n-2 location repetition effect and the contribution of inhibitory control processes when switching auditory attention in space between three locations (e.g., A, B, and C). The n-2 location repetition effect may appear either as an ABA cost, indicating that inhibitory control processes were engaged to reduce the interference from previously attended, and then abandoned, auditory locations, or as an ABA benefit, reflecting positive priming and facilitated target processing at a previously relevant location. In task switching studies, ABA costs are interpreted as evidence in favor of inhibitory control processes supporting flexible reconfiguration of task sets.

Therefore, in addition to examining age-related differences in target-distractor segregation via the well-established congruency effect, this listening task measures the n-2 location repetition effect, potentially providing a new and complementary index of inhibitory control during auditory attention switching. We did so while controlling for sensory hearing capacity to ensure that any potential age-related differences in listening performance reflect cognitive rather than peripheral auditory factors.

## Methods

### Transparency and openness

We report how the sample size was determined, along with all data exclusions, manipulations, and measures in the study, and we follow the Journal Article Reporting Standards (JARS, Appelbaum et al., 2018). All deidentified data and analysis code are openly available. This study’s design and its analysis were not preregistered. Analyses were performed using RStudio (version 4.3.0, R Core Team, 2023).

### Stimuli

At the beginning of each trial, a black arrow (5 cm) was visually presented at the centre of the screen, indicating the to-be-attended sound source (left, right, or front). The auditory stimuli were spoken digits from 1 to 9 (excluding 5), pronounced by two male and two female speakers in an anechoic chamber at the Institute of Technical Acoustics at RWTH Aachen University. All audio files were sampled at 44.1 kHz with 16-bit resolution and maintained a signal-to-noise ratio of above 60 dB (see Oberem & Fels, 2020, for a technical report on the speech material). All utterances from the four speakers were edited to 600 ms, time-aligned based on the onset of the first vowel, and normalised for intensity and pitch while preserving natural gender differences. Stimuli were presented as two-speaker pairs (never the same digit), always one male and one female voice, to ensure a clear distinction. In each trial, a target and a distractor digit were played simultaneously at 75 dB from two of three possible locations.

### Procedure

Participants initially completed two screening procedures to ensure their eligibility for participation. The DemTect test (Kalbe et al., 2004) was administered to identify possible mild cognitive impairment, followed by Pure-Tone Audiometry (PTA), which, according to WHO guidelines (Chadha et al., 2021), reliably assesses sensory hearing capacity by means of hearing thresholds. Together, these procedures took approximately 25 min.

The cued selective listening task was implemented in PsychoPy (version 2023.2.3, Peirce et al., 2022). Participants sat 70 cm from a 17-inch monitor (Samsung SyncMaster 940B) and were surrounded by three Genelec 8010AP loudspeakers positioned 140 cm from the head and 115 cm above the floor: one frontal speaker (0°) above the monitor and two lateral speakers angled ± 45°. Task instructions emphasised speed and accuracy. Participants categorised the digit presented at the cued location as smaller or larger than 5, pressing one of two keyboard keys (left for < 5, right for > 5), with fixed magnitude-response mapping^1^ to mirror the left-to-right mental number line (Dehaene et al., 1993).

Before the main task, participants completed several practice blocks. Three initial blocks presented single stimuli from one location (left, right, or front) per block to familiarise participants with all spoken digits and the stimulus-response mapping. Each of these blocks consisted of five trials. A fourth block introduced trial-to-trial switching between cued locations, with only a single target stimulus being presented at a time. Two additional blocks introduced simultaneous target-distractor presentation: first with a cue-stimulus interval (CSI) of 1000 ms and a response-cue interval (RCI) of 1000 ms, and subsequently with the timing used in the actual experiment (CSI = 500 ms; RCI = 1000 ms). The last three practice blocks each consisted of 10 trials. An experimenter was present throughout practice to ensure participants clearly understood the task requirements.

The main experiment consisted of 15 blocks of 50 trials each, separated by short breaks. Each trial began with a visual cue indicating the target location. After 500 ms, two spoken digits were presented simultaneously from two of the three different loudspeakers. The cue remained visible until the key press. Incorrect responses triggered a 500 ms error message (“Fehler!”), with non-responses after 3000 ms also counting as errors.

### Design

The study employed a mixed factorial design. N-2 location sequence (ABA vs. CBA) and congruency (congruent vs. incongruent) served as within-subject independent variables. In congruent trials, the target and distractor digits belonged to the same magnitude category (both < 5 or both > 5), eliciting the same response. In incongruent trials, target and distractor digits belonged to different magnitude categories (e.g., target > 5, distractor < 5, or vice versa), requiring different responses. Participants’ age group (younger vs. older adults) was included as a between-subject independent variable. The dependent variables were reaction time (RT) and error rate (ER). To examine the influence of age-related sensory decline, each participant’s hearing capacity was calculated from individual audiograms.

The first two trials of each block were excluded from the counterbalancing of conditions, as the n-2 location sequence could not be computed. The remaining trials were evenly distributed across conditions within each experimental block. This counterbalancing procedure resulted in a total of 360 ABA and 360 CBA trials per participant, with an equal number of congruent and incongruent trials in each sequence type. Within each block, each target location appeared equally often across the three loudspeaker positions for all combinations of n-2 location sequence and congruency. Trial sequences were pseudorandomised to ensure that the target location from trial n-1 did not reappear as the target in trial n, and similarly, that the distractor location from trial n-1 did not reappear as the distractor in trial n. Additionally, to control for n-1 priming effects, further constraints ensured that, across ABA and CBA trials, an equal number of trials in which the previous (n-1) target became the current distractor, and the previous (n-1) distractor became the current target. Consequently, given the three loudspeaker positions, when a target location was repeated relative to the n-2 trial, the corresponding distractor location was also repeated.

An a-priori power analysis was conducted using G*Power software (Faul et al., 2007). To test the predictions derived from the inhibitory deficit hypothesis, the primary effects of interest concerned age-related differences in auditory attention switching (n-2 location repetition effect) and target-distractor segregation (congruency effect). Accordingly, we operationalised the interactions age group × n-2 location sequence and age group × congruency as between-group difference scores, allowing a power analysis based on an independent-samples t-test (two-tailed, α = 0.05). This approach represents a simplification of the full within- and between-subject factorial design. We powered the study to detect a medium-sized effect (*d* = 0.60) as the minimum effect of interest. This choice was guided by the expectation that, once individual differences in hearing abilities are accounted for, age-related differences in attentional control would be at least of moderate magnitude. With 80% power (1 − β = 0.80), the analysis indicated a required total sample size of *n* = 90 (45 per group). Subsequent evidence using closely related auditory paradigms (e.g., Falanga et al., 2026) reported larger age-related differences in auditory attention switching ($\:{{\upeta\:}}_{\mathrm{p}}^{2}$ = 0.15, corresponding to Cohen’s *d* ≈ 0.84), suggesting that the present design was adequately powered to detect the age-related effects of interest.

### Participants

**Fig. 1:**
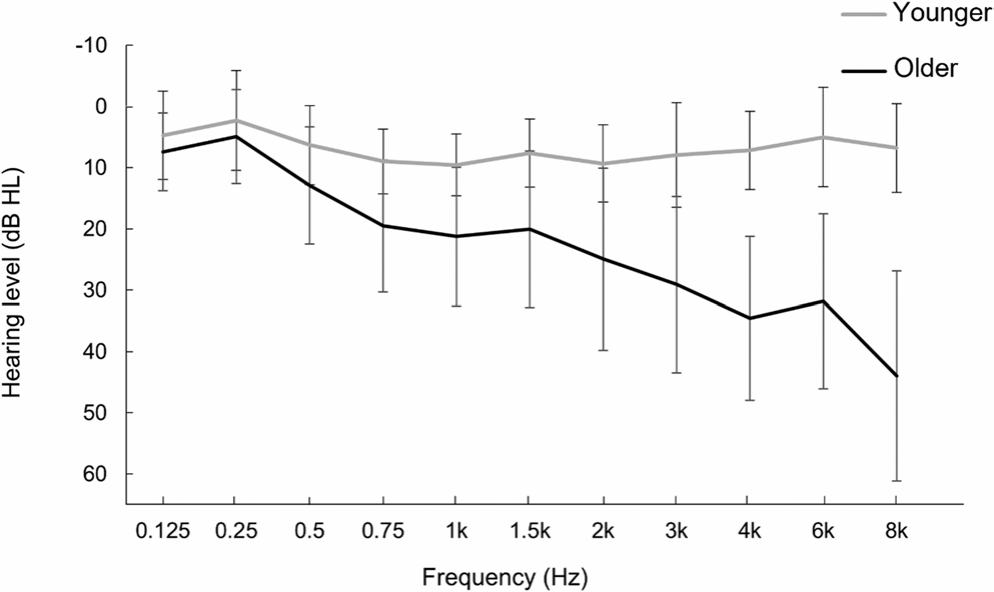
Composite Audiograms of Younger and Older Participants. Note. Line graph of mean hearing thresholds (dB HL) for younger and older adults, ranging from 125 Hz to 8 kHz. Younger adults show on average non-pathological hearing thresholds across all frequencies. Older adults show elevated hearing thresholds at mid-to-high frequencies. Error bars represent standard errors

**Fig. 2:**
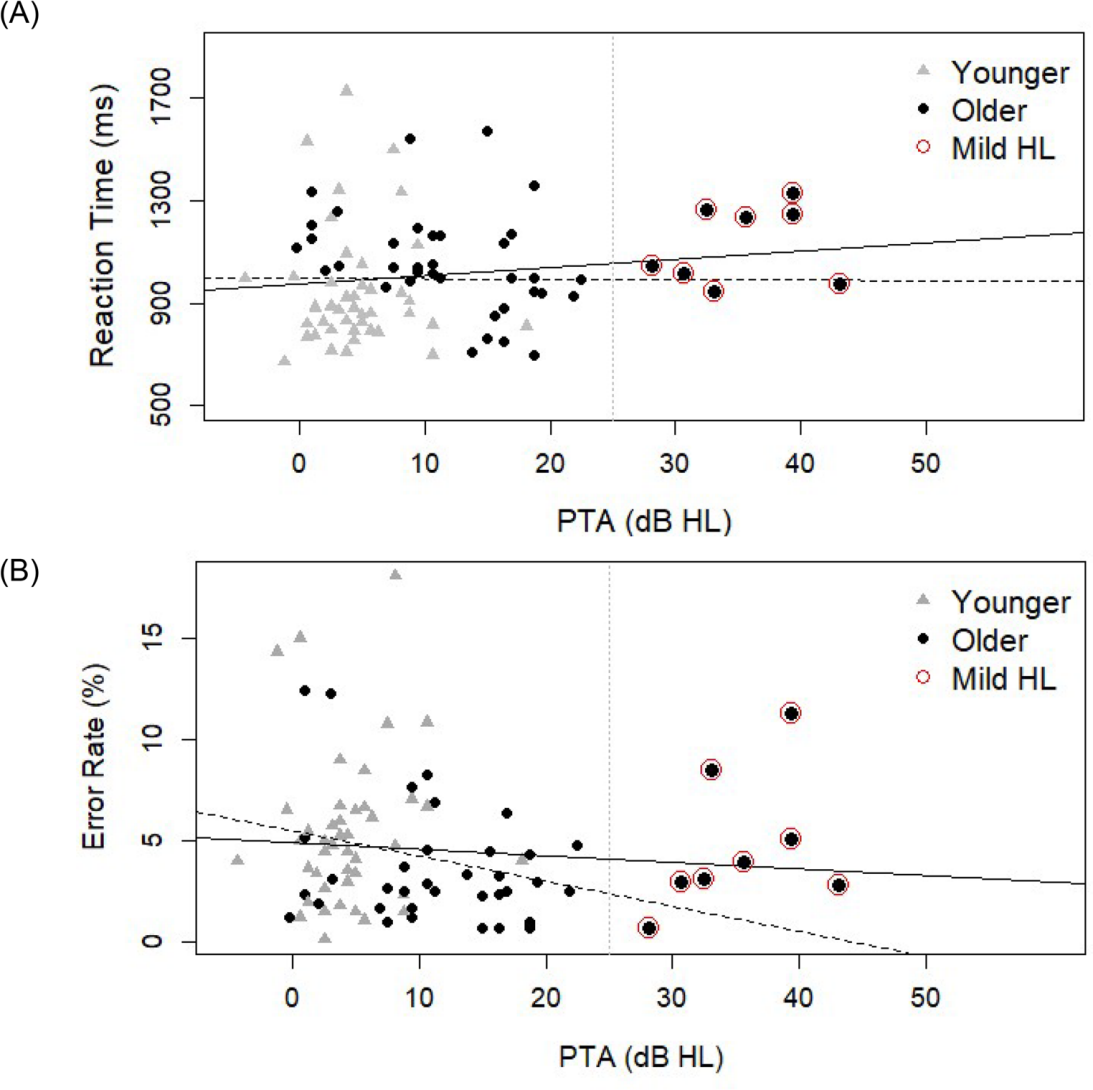
Scatterplots Illustrating the Relationship Between Pure-Tone Average (PTA) in Decibels (dB HL) and Listening Performance Measures (RT and ER) Across Age Groups (Younger and Older). Note. Panel **A** depicts RT, and Panel **B** depicts ER. Vertical dotted lines indicate the 25 dB HL threshold differentiating normal hearing from hearing impairment (per WHO guidelines). Data points for participants with mild hearing impairment are marked with a red circle (*n* = 8; labelled “Mild HL”). Two linear regression lines are shown in each plot: the solid line represents the correlation across the entire sample, while the dashed line represents the relationship excluding participants with mild hearing impairment. Although excluding these participants suggested a negative association between PTA and ER, *r* = − .22, *t*(78) = -1.98, *p* = .051, this trend did not reach significance and should be interpreted with caution

A total of 90 participants completed the experiment: 45 younger adults (age 19–26 years; *M* = 22.0 years, *SD* = 1.8 years; 9 males, 2 diverse) and 45 older adults (age 61–80 years; *M* = 68.0 years, *SD* = 3.8 years; 15 males). Hearing thresholds were assessed using ascending PTA for frequencies ranging from 125 Hz to 8 kHz. Figure 1 presents the mean audiograms for both age groups, illustrating age-related differences in sensory hearing abilities. According to the WHO (Chadha et al., 2021) criteria for classifying hearing impairment, all younger participants exhibited normal hearing capacity (i.e., ≤ 25 dB HL). In contrast, older participants showed elevated thresholds, especially at higher frequencies (> 4 kHz). Eight older participants (see Fig. 2) met WHO criteria for mild hearing impairment (Chadha et al., 2021). All procedures were conducted in accordance with the Declaration of Helsinki and were approved by the Ethics Committee of the University Hospital Aachen (EK 24–429); written informed consent was obtained from all participants.

## Results

For both RT and ER analyses, practice trials and trials with RTs < 200 ms or > 3000 ms were removed. In addition, the first two trials of each block were excluded because the n-2 repetition sequence could not be classified as ABA or CBA trials. In RT analyses, error trials and the two subsequent trials were excluded, while in ER analyses, only the two post-error trials were excluded, as these could not be correctly classified as either ABA or CBA trials. Outliers were further removed by discarding trials with RTs exceeding ± 3 standard deviations (*SDs*) from each participant’s mean within each condition (0.75% for younger and 0.65% for older adults). Data from one older and one younger participant were excluded from the main analysis because their ER exceeded the overall mean ER by more than three *SDs*, suggesting possible difficulties in understanding the task instructions.

In line with WHO guidelines (Chadha et al., 2021), a single hearing capacity value was obtained for each participant, averaging hearing thresholds across four frequencies (500 Hz, 1 kHz, 2 kHz, and 4 kHz) and across both ears. Following the outlier exclusion procedure outlined above, younger adults showed a mean PTA of 4.37 dB HL (*SD* = 3.78 dB HL), whereas older adults showed a mean PTA of 15.80 dB HL (*SD* = 11.14 dB HL). To assess a potential relationship between hearing capacity and overall listening performance, Pearson correlations were computed between individual PTA values and mean RT and ER (Fig. 2). These correlation analyses revealed no significant relationship between PTA and RT, *r* = .15, *t*(86) = 1.37, *p* = .176, or between PTA and ER, *r* = − .09, *t*(86) = − 0.86, *p* = .392. Overall, these results suggest that individual differences in non-pathological hearing capacity did not influence listening performance in the current task. 

To further assess the impact of age-related sensory decline on listening performance, individual PTA values were centred on the sample mean and included as a continuous covariate in additional analyses of covariance (ANCOVAs) conducted on both RT and ER (see Appendix). Overall, the contribution of non-pathological changes in hearing capacity to the control of auditory attention was limited in the present study. A two-way interaction emerged in the ER analysis, indicating a significant relationship between PTA and the congruency effect; however, this effect should be interpreted with caution as PTA showed low and nonsignificant correlations with overall ER (Fig. 2B).

Two separate three-way mixed analyses of variance (ANOVAs) were conducted on mean RT and ER, with age group (younger vs. older) as an independent between-subject variable, n-2 location sequence (ABA vs. CBA), and congruency (incongruent vs. congruent) as independent within-subject variables. To account for general slowing in older adults (Salthouse, 1996) and to reduce the influence of baseline speed differences on the shape of RT distributions, the same ANOVA was conducted using log-transformed RT. This logarithmic transformation, which is widely used in aging research (Hirsch et al., 2016; Kray & Lindenberger, 2000; Mayr, 2001), was applied to ensure more comparable distributions across groups. Similarly, another ANOVA was carried out on arcsine square root transformed ER data to stabilise the variances of proportion data (Winer, 1962). Only main effects and interactions that differed from those observed in the raw RT or raw ER analyses are reported.

Finally, to account for potential speed-accuracy trade-offs, we conducted an additional analysis using the same ANOVA structure as in the primary analyses, but including the Linear Integrated Speed-Accuracy Score (LISAS; Vandierendonck, 2017) as a dependent variable. This analysis yielded a pattern of results consistent with the separate analyses of RT and ER (see Supplementary Materials). We also conducted exploratory analyses to examine whether stimulus-response spatial compatibility (i.e., compatible, incompatible, or undefined for centrally presented targets) influenced overall listening performance or modulated age-related differences. Previous work using a similar cued selective listening paradigm reported slower responses when stimulus location was incompatible with the response side, but no interaction with attentional switch costs (Lawo and Koch 2014a). In the present study, compatibility influenced overall RT and interacted with n-2 location sequence, but it did not modulate the age-related pattern of the n-2 location repetition effect (see Supplementary Materials).

### Reaction time

**Fig. 3:**
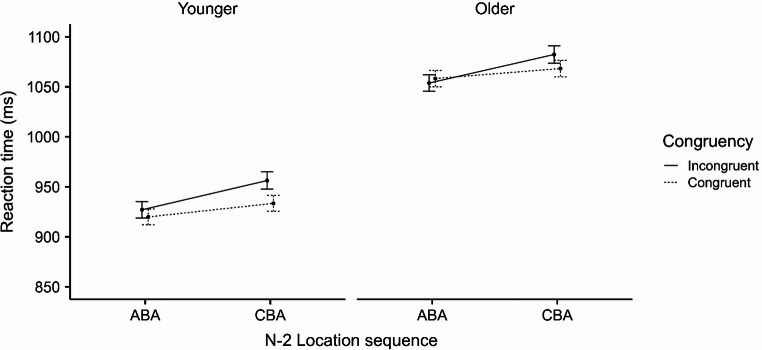
Mean Reaction Times (ms) as a Function of n-2 Location Sequence (ABA vs. CBA), Congruency (Incongruent vs. Congruent), Age Group (Younger vs. Older). Note. Error bars represent 95% confidence intervals

The ANOVA on RT revealed a significant main effect of age group, *F*(1, 86) = 7.85,*p* = .006, $\:{{\upeta\:}}_{\mathrm{p}}^{2}$ = 0.08, indicating longer RT for older participants (1066 ms) than for younger participants (934 ms). The main effect of n-2 location sequence was significant, *F*(1, 86) = 37.13, *p* < .001, $\:{{\upeta\:}}_{\mathrm{p}}^{2}$ = 0.30, indicating shorter RT on ABA trials (991 ms) than on CBA trials (1011 ms), corresponding to a mean n-2 location repetition effect of 20 ms (i.e., n-2 location repetition positive priming). The two-way interaction between age group and n-2 location sequence was nonsignificant, *F* < 1 (see Fig. 3).

A significant main effect of congruency was observed, *F*(1, 86) = 12.81, *p* = .001, $\:{{\upeta\:}}_{\mathrm{p}}^{2}$ = 0.13, indicating longer RT on incongruent trials (1006 ms) compared to congruent trials (996 ms), corresponding to a congruency effect of 10 ms. The two-way interaction between congruency and age group was nonsignificant, *F*(1, 86) = 3.19, *p* = .077, $\:{{\upeta\:}}_{\mathrm{p}}^{2}$ = 0.04. However, this interaction was significant when using log-transformed RT, *F*(1, 86) = 4.65, *p* = .034,$\:{{\upeta\:}}_{\mathrm{p}}^{2}$ = 0.05, suggesting a smaller congruency effect for older compared to younger participants (5 ms vs. 15 ms, respectively).

The two-way interaction between n-2 location sequence and congruency was significant, *F*(1, 86) = 7.12, *p* = .009, $\:{{\upeta\:}}_{\mathrm{p}}^{2}$ = 0.08, indicating a reduced congruency effect and thus facilitated processing during ABA trials (1 ms) relative to CBA trials (19 ms). Notably, the three-way interaction between age group, n-2 location sequence, and congruency was nonsignificant, *F* < 1 (see Fig. 3). Such a consistent pattern across two independent age groups suggests that the observed two-way interaction between congruency and n-2 location sequence represents a stable feature in the control of auditory attention that is not affected by age.

### Error rate

The ANOVA on ER revealed a significant main effect of age group, *F*(1, 86) *=* 4.38,*p* = .039, $\:{{\upeta\:}}_{\mathrm{p}}^{2}$ = 0.05, indicating a higher ER for younger compared to older participants (5.0% vs. 3.6%, respectively). The main effect of n-2 location sequence was nonsignificant, *F*(1, 86) = 1.43, *p* = .235, $\:{{\upeta\:}}_{\mathrm{p}}^{2}$ = 0.02, suggesting neither a positive priming nor additional costs in target processing when returning to a previously attended location. However, the two-way interaction between age group and n-2 location sequence was significant, *F*(1, 86) = 4.26, *p* = .042, $\:{{\upeta\:}}_{\mathrm{p}}^{2}$ = 0.05, suggesting that while younger adults’ performance was similar across ABA and CBA trial types, older adults showed more accurate performance on ABA relative to CBA trial types (i.e., n-2 location repetition positive priming).

**Fig. 4:**
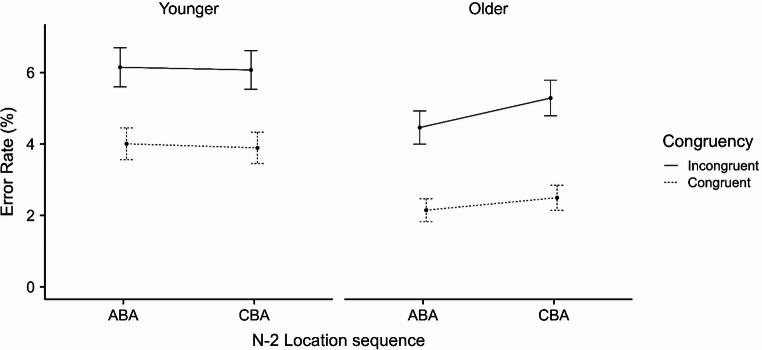
Mean Error Rate (%) as a Function of n-2 Location Sequence (ABA vs. CBA), Congruency (Incongruent vs. Congruent), Age Group (Younger vs. Older). Note. Error bars represent 95% confidence intervals

A significant main effect of congruency was observed, *F*(1, 86) = 61.24, *p* < .001, $\:{{\upeta\:}}_{\mathrm{p}}^{2}$ = 0.42, indicating increased ER on incongruent trials (5.5%) compared to congruent trials (3.1%), corresponding to a congruency effect of 2.4%. The two-way interaction between congruency and age group was nonsignificant, *F* < 1. The two-way interaction between n-2 location sequence and congruency was also nonsignificant, *F* < 1. Finally, the three-way interaction between age group, n-2 location sequence, and congruency was nonsignificant, *F* < 1 (see Fig. 4). No differences in main effects or interactions emerged when conducting the same ANOVA using arcsine square root transformed ER.

## Discussion

Using an extended version of the cued selective listening task introduced by Koch et al. (2011), we investigated whether inhibitory deficits (Hasher & Zacks, 1988) contribute to age-related changes in the control of auditory attention. Specifically, we assessed, in a multi-talker listening environment, two behavioural markers of inhibitory control: the congruency effect, reflecting target-distractor segregation (Kiesel et al., 2010), and the n-2 location repetition effect, assessing the contribution of inhibitory control processes during auditory attention switching (Koch et al., 2010). In addition, we examined whether reduced sensory hearing capacity constrains selective listening performance under challenging listening conditions (Caso et al., 2024; Falanga et al., 2026).

Consistent with prior studies employing a similar selective listening paradigm (Falanga et al. 2026; Lawo and Koch 2014b; Oberem et al. 2017), older adults showed general slowing (Salthouse, 1996). However, older adults’ performance was generally more accurate relative to that of younger adults, suggesting a more cautious response strategy and a speed-accuracy trade-off (Schuch, 2016). These findings indicate that although older adults with non-pathological hearing loss show slower sensory processing and delayed responses, auditory attention remains largely preserved (Caso et al., 2024; Falanga et al., 2026). Importantly, we did not observe a linear relationship between sensory hearing capacity and general listening performance (RT and ER), indicating that interindividual variability in hearing thresholds did not influence overall task performance (see Fig. 2).

A central finding of this study concerns the ability to segregate target and distractor information in complex multi-talker environments. The congruency effect investigated here (Kiesel et al., 2010; Monsell et al., 2019) served as an implicit measure of target-distractor segregation, reflecting, at least in part, the ability to inhibit auditory distractors and to resist interference. Consistent with earlier findings (Koch et al., 2011; Nolden et al., 2019), participants were slower and more error-prone on incongruent than on congruent trials, suggesting failures in target-distractor segregation and processing of distractor information despite being irrelevant for the task (Kiesel et al., 2010).

However, no robust age-related differences were found in the congruency effect (Lawo and Koch 2014b; Oberem et al. 2017), indicating that target-distractor segregation was not reduced with age under these listening conditions. A small difference emerged when log-transformed values were analysed: older adults showed a slightly reduced congruency effect relative to younger adults. In line with the speed-accuracy trade-off observed in older adults’ listening performance, this pattern may reflect a more cautious response strategy, which could have in turn increased resistance to distractor interference. Taken together, this pattern indicates that older adults with non-pathological hearing loss can efficiently segregate competing auditory streams (Caso et al., 2024; Falanga et al., 2026), performing similarly to young normal-hearing adults.

Another important contribution of this study concerns the n-2 location repetition effect. In the present study, unlike previous selective listening tasks (Koch et al., 2011; Monsell et al., 2019), which manipulated target selection criteria relative to the previous (n-1) trial to assess auditory switch costs, we manipulated the cued location relative to the n-2 trial (ABA vs. CBA trial) in order to assess the n-2 location repetition effect. This effect served as a proxy for the n-2 repetition costs (i.e., difference between ABA and CBA trials) often observed in task-switching studies in which participants switch between three different tasks (Lawo et al., 2012; Mayr & Keele, 2000; Schuch, 2016). In classical task-switching paradigms, performance is typically worse when returning to a recently abandoned task (ABA) compared to switching to a new one (CBA). A performance decline observed during ABA relative to CBA trials is thought to reflect persisting inhibition of the previously relevant task (see Koch et al., 2010). By analogy, employing a listening setup with three different sound sources, we expected that attending to a location that was relevant two trials back (ABA) might also impair performance due to inhibition applied at that location.

Our results revealed an n-2 location repetition benefit: performance was facilitated on ABA trials compared to CBA trials, but only in RT. This pattern suggests that alternative control mechanisms, rather than persisting inhibition of previously attended location or inhibition of return, may exert a stronger influence during auditory attention switching. Three non-mutually exclusive accounts may explain the observed n-2 location repetition benefit. First, residual activation of previously attended location may facilitate target processing when that location becomes relevant again, consistent with positive priming accounts (e.g., Maljkovic & Nakayama, 1994). Second, facilitation may arise through retrieval processes tied to the specific target-distractor configuration presented in trial n-2 (see Design). If these spatial target-distractor contingencies are learned, returning to the same target location two trials later may retrieve the previously associated distractor location, thereby reducing competition and facilitating target selection. Third, persisting inhibition of distractor locations could also contribute, because repetitions of a target location coincide with repetitions of a distractor location, inhibition of the distractor may carry over and indirectly benefit target selection, independently of any facilitation related to the target itself.

Crucially, no age-related differences emerged in the n-2 location repetition effect for RT. Thus, our findings suggest that younger and older adults generally exhibit comparable efficiency in the cognitive control processes responsible for switching auditory attention in space. However, an age-related difference in the n-2 location repetition effect was observed in ER: while younger adults showed neither n-2 location repetition benefit nor cost, older adults made fewer errors on ABA than on CBA trials. Importantly, this older adults’ repetition benefit in ER aligns in direction with the overall ABA benefit observed in RT across both age groups. Although our n-2 manipulation differs from the location switch vs. repeat contrast used by Falanga et al. (2026), the ER data similarly indicate an age-related modulation of accuracy: only older adults’ listening performance was more accurate when returning to a previously attended location (ABA) than when switching to a new one (CBA). Similarly, Falanga et al. (2026) reported larger switch costs for older adults relative to younger adults. However, in the previous version of cued selective listening tasks (Falanga et al. 2026; Koch et al. 2011; Lawo and Koch 2014b), the target location sequence was manipulated relative to the immediately preceding trial. Therefore, the carry-over from the n-1 trial may have been generally stronger, amplifying conflict between competing attention sets when a switch in attention is required, specifically in older adults. In the present design, with sequences controlled over a longer time, carry-over effects from n-2 trials were likely reduced in both age groups. The small age-related difference we observed may therefore reflect, in the group of older adults, a slower reconfiguration of previously relevant attention sets, resulting in facilitation when the location repeats but not when it switches.

Alternatively, in younger adults’ ER, the combined influence of facilitatory and inhibitory control mechanisms at play during attention set reconfiguration may cancel out any observable n-2 repetition effect. Older adults, by contrast, may rely more on the facilitatory component, which raises the possibility that inhibitory contributions to attention set reconfiguration get comparatively weaker with increasing age (Hasher & Zacks, 1988; Mayr, 2001; Schuch, 2016). However, the present data do not provide evidence of an inhibitory deficit per se (see Rey-Mermet & Gade, 2018, for a review). Although modest in size, the findings point to subtle age-related changes in cognitive control during auditory attention switching, without implying a fundamental impairment of inhibitory control processes. Notably, this age-related modulation was primarily observed in ER and was not clearly reflected in the integrated LISAS measure (see Supplementary Materials).

Finally, congruency interacted with the n-2 location sequence in RT. Across both age groups, the congruency effect was reduced during ABA compared to CBA trials, suggesting that returning to a previously attended location improved target-distractor segregation in both age groups. Although the present paradigm does not allow us to isolate whether this reflects target facilitation, distractor inhibition, or both, the interaction provides converging evidence that processing a target in a previously attended location supports more efficient selective listening performance in such a multi-talker setup.

Taken together, our findings suggest that while older adults exhibit general slowing and may rely more strongly on facilitatory processes when switching auditory attention in space, their ability to segregate competing auditory input and to resist distractor interference remains largely intact. This pattern differs from the age-related switch-specific deficit reported by Falanga et al. (2026). One possible explanation concerns the difference in sequence structure across paradigms. While Falanga et al. compared n-1 repeat and switch trials in a two-location setup, the present task used a three-location setup and required participants to switch attention on every trial due to the sequence structure required to compute the n-2 location repetition effect. In this “switch-only” context, the absence of direct n-1 repeat trials may have promoted a sustained switch-ready control state, thereby reducing the performance difference between n-2 repeat (ABA) and switch (CBA) trials and attenuating observable age-related differences in auditory attention switching. Ultimately, the present study provides no evidence for an age-related inhibitory deficit in auditory attention switching and target-distractor segregation.

## Conclusion

The present study investigated age-related decline in auditory attention using a cued selective listening task. Older adults showed general slowing but maintained accuracy, suggesting preserved attentional control. Importantly, no evidence emerged for an age-related inhibitory deficit in either auditory attention switching or target-distractor segregation. Small differences in n-2 location repetition effects likely reflect subtle age-related changes during attention set reconfiguration rather than impaired inhibition. Overall, these findings indicate that auditory attention remains largely intact in healthy aging, with older adults effectively resisting competing auditory information under complex listening conditions.

## Supplementary Information

Below is the link to the electronic supplementary material.

Supplementary Material 1

## Data Availability

The de-identified data and analysis code are available at the following stable links: https://doi.org/10.23668/psycharchives.21902, https://doi.org/10.23668/psycharchives.21901.

## Appendix

### Analyses of covariance (ANCOVAs)

The impact of sensory decline on listening performance was investigated using Pure-Tone Audiometry (PTA); PTA values were centred on the sample mean and included as a continuous covariate in two separate mixed ANCOVAs on mean RT and ER. However, because PTA showed low and nonsignificant correlations with RT and ER (Fig. 2), its role as a covariate should be interpreted cautiously. The between-subject factor was age group (younger vs. older), while the within-subject factors were congruency (incongruent vs. congruent) and n-2 location sequence (ABA vs. CBA).

### Reaction time

The ANCOVA on RT revealed a significant main effect of age group, *F*(1, 85) = 5.85,*p* = .018, $\:{{\upeta\:}}_{\mathrm{p}}^{2}$ = 0.06, with older participants responding more slowly (1066 ms) than younger participants (934 ms). Including hearing thresholds (i.e., PTA) as a continuous covariate did not significantly affect RT, *F* < 1.

The main effect of n-2 location sequence was significant, *F*(1, 85) = 36.71, *p* < .001, $\:{{\upeta\:}}_{\mathrm{p}}^{2}$ = 0.30, with faster responses on ABA trials (991 ms) than on CBA trials (1011 ms), yielding a mean n-2 location repetition effect of 20 ms. The interaction between age group and n-2 location was nonsignificant, *F* < 1, as was the interaction of PTA and n-2 location sequence, *F* < 1, indicating that neither age nor PTA modulated the n-2 location repetition effect.

A significant main effect of congruency was also found, *F*(1, 85) = 13.09, *p* = .001, $\:{{\upeta\:}}_{\mathrm{p}}^{2}$ = 0.13, indicating longer RT on incongruent trials (1006 ms) relative to congruent trials (996 ms), corresponding to a congruency effect of 10 ms. Both the interaction between congruency and age group, *F* < 1, and the interaction of PTA and congruency did not reach significance, *F*(1, 85) = 2.85, *p* = .095, $\:{{\upeta\:}}_{\mathrm{p}}^{2}$ = 0.03, indicating that the congruency effect was not influenced by age or PTA.

The interaction between n-2 location sequence and congruency was significant, *F*(1, 85) = 7.05, *p* = .009, $\:{{\upeta\:}}_{\mathrm{p}}^{2}$ = 0.08, indicating a reduced congruency effect and thus selective processing during ABA trials (1 ms), relative to CBA trials (19 ms). Finally, both the three-way interaction between age group, n-2 location sequence, and congruency, *F* < 1, and the three-way interaction including PTA, n-2 location sequence, and congruency, *F* < 1, were nonsignificant. No differences in main effects or interactions emerged when conducting the same ANCOVA using log-transformed RT.

### Error rate

The ANCOVA on ER revealed an effect of age group that marginally approached significance, *F*(1, 85) *=* 3.68, *p* = .058, $\:{{\upeta\:}}_{\mathrm{p}}^{2}$ = 0.04. However, when using arcsine square root-transformed ER, the effect became significant, *F*(1, 85) *=* 5.67, *p* = .019, $\:{{\upeta\:}}_{\mathrm{p}}^{2}$ = 0.06, with younger adults showing higher ER than older adults (5.0% vs. 3.6%, respectively). PTA did not significantly affect ER, *F* < 1.

The main effect of n-2 location sequence was nonsignificant, *F* < 1, suggesting no overall n-2 location repetition effect in ER. The two-way interaction between age group and n-2 location sequence was nonsignificant, *F*(1, 85) = 2.61, *p* = .110, $\:{{\upeta\:}}_{\mathrm{p}}^{2}$ = 0.03. However, this interaction became significant when using arcsine square root-transformed ER, *F*(1, 85) = 6.48, *p* = .013, $\:{{\upeta\:}}_{\mathrm{p}}^{2}$ = 0.07, indicating as in the main analysis, that only older adults showed a better performance during ABA relative to CBA trial types (i.e., n-2 location repetition positive priming). The interaction between PTA and n-2 location sequence was nonsignificant, F < 1.

A significant main effect of congruency was observed, *F*(1, 85) = 43.90, *p* < .001, $\:{{\upeta\:}}_{\mathrm{p}}^{2}$ = 0.34, with higher ER on incongruent trials (5.5%) compared to congruent trials (3.1%), corresponding to a congruency effect of 2.4%. The interaction between congruency and age group was nonsignificant, *F*(1, 85) = 3.32, *p* = .072, $\:{{\upeta\:}}_{\mathrm{p}}^{2}$ = 0.04, but became significant with arcsine square root-transformed ER, *F*(1, 85) = 5.13, *p* = .026, $\:{{\upeta\:}}_{\mathrm{p}}^{2}$ = 0.06, indicating a larger congruency effect for older relative to younger adults (2.6% vs. 2.2%, respectively). The interaction between congruency and PTA was significant, *F*(1, 85) = 4.21, *p* = .043, $\:{{\upeta\:}}_{\mathrm{p}}^{2}$ = 0.05, and approached significance with arcsine square root-transformed ER, *F*(1, 85) = 3.48, *p* = .066, $\:{{\upeta\:}}_{\mathrm{p}}^{2}$ = 0.04. Thus, this effect should be interpreted with caution as it was not robust across transformations. Taken together, the two-way interactions involving congruency with age groups and PTA were modest in size and not robust across transformations. Thus, these interaction effects should be interpreted with caution.

The two-way interaction between n-2 location sequence and congruency was also nonsignificant, *F* < 1. Finally, both the three-way interaction between age group, n-2 location sequence, and congruency, *F* < 1, and the three-way interaction including PTA, n-2 location sequence, and congruency, *F* < 1, were nonsignificant.

## Public significance statement

Older adults often face challenges in understanding speech in multi-talker environments. Previous studies have suggested that, in addition to sensory decline, age-related deficits in inhibitory control might increase these listening difficulties. Our study shows that, despite general slowing, healthy older adults can efficiently focus on a target speaker, ignore distractions, and switch attention between different sound sources, similar to younger adults. These findings suggest that auditory attention remains largely preserved in healthy aging.

## Footnotes

1 Because responses were lateralised (left key for < 5; right key for > 5) and target stimuli were presented either from a left, central or right location, the present design could in principle give rise to stimulus-response spatial compatibility (Simon-like) effects. In addition, because each numerical magnitude was mapped to one response side and this mapping was kept constant across participants, any spatial-numerical compatibility (SNARC-like) effects cannot be disentangled from stimulus-response spatial compatibility in the present design.
