# Supplementary material for: Exploring age-related inhibitory deficits in auditory attention: Evidence from attention switching
Source: Psychol Res. 2026 May 11;90(3):88. doi: 10.1007/s00426-026-02306-5 (PMC13161260; doi:10.1007/s00426-026-02306-5)
Supplement: Supplementary file 1 — Supplementary Material 1 [file 426_2026_2306_MOESM1_ESM.docx]

**Title:** Age-related inhibitory deficits in auditory attention: Evidence from attention switching

**Journal:** Psychological Research

**Authors:** Luigi Falanga, Thomas Deutsch, Janina Fels, Klaus Willmes, Denise N. Stephan, and Iring Koch

**Corresponding author:** Luigi Falanga, Institute of Psychology, Chair of Cognitive and Experimental Psychology

**Email:** [luigi.falanga@psych.rwth-aachen.de](mailto:luigi.falanga@psych.rwth-aachen.de)

# **Supplementary Materials**

**S1. Linear Integrated Speed-Accuracy Score (LISAS)**

To account for potential speed-accuracy trade-offs, we conducted an additional analysis using the Linear Integrated Speed-Accuracy Score (LISAS). LISAS combines RT and ER into a single performance measure by adding a penalty for errors scaled by the relative variability of RT and ER (Vandierendonck, 2017). The scaling factor was computed using the overall standard deviations of RT and ER across all trials and participants, resulting in a constant penalty applied equally across conditions and age groups. Raw RT values were used to maintain LISAS as a time-based performance measure. Trial inclusion and outlier exclusion followed the same criteria used for the ER analyses. The resulting LISAS values were analysed using the same ANOVA structure as in the primary analyses, with age group (younger vs. older) as a between-subjects factor and n-2 location sequence (ABA vs. CBA) and congruency (incongruent vs. congruent) as within-subject factors.

The ANOVA on LISAS revealed a trend toward a significant main effect of age group, *F*(1, 86) = 3.85, *p* = .053, $\eta_{p}^{2}$ = .04, although descriptively LISAS scores were higher for older participants (1141 ms) than for younger participants (1033 ms). The main effect of n-2 location sequence was also significant, *F*(1, 86) = 18.74, *p* < .001, $\eta_{p}^{2}$ = .18, indicating lower LISAS scores on ABA trials (1075 ms) than on CBA trials (1100 ms), corresponding to an n-2 location repetition benefit of 25 ms. The two-way interaction between age group and n-2 location sequence was nonsignificant, *F*(1, 86) = 1.87, *p* = .175, $\eta_{p}^{2}$ = .02, indicating that the n-2 location repetition effect was comparable across age groups.

The main effect of congruency was significant, *F*(1, 86) = 61.00, *p* < .001, $\eta_{p}^{2}$ = .41, reflecting higher LISAS scores for incongruent trials (1115 ms) compared to congruent trials (1060 ms), corresponding to a congruency effect of 55 ms. The interaction between age group and congruency was nonsignificant, *F* < 1. The interaction between n-2 location sequence and congruency was nonsignificant, *F*(1, 86) = 3.82, *p* = .054, $\eta_{p}^{2}$ = .04, although descriptively the congruency effect differed between ABA and CBA trials. Finally, the three-way interaction between age group, n-2 location sequence, and congruency was nonsignificant, *F* < 1. Overall, the LISAS analysis closely mirrored the pattern observed in the separate RT and ER analyses, indicating robust congruency and sequence effects when speed and accuracy were considered jointly, while providing no evidence for reliable age-related modulation of the n-2 location repetition effect.

**S2. Stimulus-response spatial compatibility**

To examine whether compatibility between target location and required response influenced listening performance, we conducted an exploratory analysis of stimulus-response spatial compatibility effects. Because responses in the present task were lateralised (left key for digits < 5; right key for digits > 5) and target stimuli could be presented from the left, centre, or right location, the design could in principle give rise to stimulus-response spatial compatibility (Simon-like) effects. We therefore created a compatibility factor with three levels: compatible (left target-left response; right target-right response), incompatible (left target-right response; right target-left response), and undefined (centrally presented targets). Exploratory analyses of variance (ANOVAs) were conducted on RT and ER using the same structure as in the primary analyses, with age group (younger vs. older) as a between-subjects factor and n-2 location sequence (ABA vs. CBA), congruency (incongruent vs. congruent), and compatibility (compatible vs. incompatible vs. undefined) as within-subject factors. Greenhouse-Geisser corrected degrees of freedom are reported where sphericity was violated.

## **Reaction time**

The ANOVA on RT revealed a significant main effect of age group, *F(*1, 86) = 7.83, *p* = .006, $\eta_{p}^{2}$ = .08, indicating longer RT for older participants (1066 ms) than for younger participants (934 ms). The main effect of n-2 location sequence was significant,
*F(*1, 86) = 34.39, *p* < .001, $\eta_{p}^{2}$ = .29, indicating shorter RT on ABA trials (991 ms) than on CBA trials (1011 ms), corresponding to a mean n-2 location repetition effect of 20 ms (i.e., n-2 location repetition positive priming). The two-way interaction between age group and n-2 location sequence was nonsignificant, *F* < 1.

A significant main effect of congruency was observed, *F(*1, 86) = 13.99, *p* = .001, $\eta_{p}^{2}$ = .14, indicating longer RT on incongruent trials (1006 ms) compared to congruent trials (996 ms), corresponding to a congruency effect of 10 ms. The two-way interaction between congruency and age group was nonsignificant, *F(*1, 86) = 2.57, *p* = .112, $\eta_{p}^{2}$ = .03. The two-way interaction between n-2 location sequence and congruency was significant, *F(*1, 86) = 6.01, *p* = .016, $\eta_{p}^{2}$ = .07, indicating a smaller congruency effect and thus reduced distractor interference during ABA trials (1 ms) relative to CBA trials (19 ms). The three-way interaction between age group, n-2 location sequence, and congruency was nonsignificant, *F* < 1.

Crucially, a significant main effect of compatibility was observed, *F(*1.72, 147.91) = 11.11, *p* < .001, $\eta_{p}^{2}$ = .11, indicating shorter RT on compatible trials (988 ms) relative to incompatible trials (999 ms) and undefined trials (1017 ms). The two-way interaction between age group and compatibility was nonsignificant, *F(*1.72, 147.91) = 2.70, *p* = .079, $\eta_{p}^{2}$ = .03, as well as the two-way interaction between congruency and compatibility,
*F(*1.92, 164.77) = 2.70, *p* = .072, $\eta_{p}^{2}$ = .03. However, the two-way interaction between n-2 location sequence and compatibility was significant, *F(*1.72, 147.91) = 5.90, *p* = .004,
$\eta_{p}^{2}$ = .06, indicating a larger n-2 location repetition benefit (i.e., positive priming) on compatible and incompatible trials (26 ms) than on trials with undefined compatibility (8 ms). The four-way interaction between age group, n-2 location sequence, congruency and compatibility was nonsignificant, *F(*1.94, 167.135) = 1.57, *p* = .211, $\eta_{p}^{2}$ < .01. No other higher-order interactions involving age group and compatibility were significant, *F* < 1.

## **Error rate**

The ANOVA on ER revealed a significant main effect of age group, *F(*1, 86) = 4.13, *p* = .045, $\eta_{p}^{2}$ = .05, indicating a higher ER for younger compared to older participants (5.0% vs. 3.6% respectively). The main effect of n-2 location sequence was nonsignificant, *F* < 1, suggesting neither a positive priming nor additional costs in target processing when returning to a previously attended location. The two-way interaction between age group and n-2 location sequence was nonsignificant, *F(*1, 86) = 3.52, *p* = .064, $\eta_{p}^{2}$ = .04, with a descriptive pattern suggesting that while younger adults’ performance was similar across ABA and CBA trial types, older adults showed more accurate performance on ABA relative to CBA trial types (i.e., n-2 location repetition positive priming).

A significant main effect of congruency was observed, *F(*1, 86) = 60.37, *p* < .001, $\eta_{p}^{2}$ = .41, indicating increased ER on incongruent trials (5.5%) compared to congruent trials (3.1%), corresponding to a congruency effect of 2.4%. The two-way interaction between congruency and age group was nonsignificant, *F* < 1, as well as the two-way interaction between n-2 location sequence and congruency, *F* < 1. Finally, also the three-way interaction between age group, n-2 location sequence, and congruency was nonsignificant, *F* < 1.

The main effect of compatibility was nonsignificant, *F* < 1, nor were its interactions with age group, *F(*1.90, 163.62) = 1.75, *p* = .179, $\eta_{p}^{2}$ = .02, or congruency, *F* < 1. However, the two-way interaction between n-2 location sequence and compatibility was significant, *F(*1.96, 168.69) = 3.63, *p* = .029, $\eta_{p}^{2}$ = .04, indicating a small n-2 location repetition positive priming of (.7%) on incompatible trials, but a near-zero effect on compatible and undefined trials. Finally, the three-way interaction between n-2 location sequence, congruency and compatibility was nonsignificant, *F(*1.68, 144.73) = 2.12, *p* = .132, $\eta_{p}^{2}$ = .02. No higher-order interactions involving age group and compatibility were significant, *F* < 1.

Overall, in the present study stimulus-response spatial compatibility influenced listening performance, particularly in RT, but did not modulate the central age-related patterns of the n-2 location repetition effect. Importantly, although the present three-location listening task revealed compatibility-related effects, it was not ideally suited to isolate Simon-like effects. In addition, because numerical magnitude was mapped onto response side and this mapping was held constant across participants, any spatial-numerical compatibility (SNARC-like) effects could not be disentangled from stimulus-response spatial compatibility in the present design. Accordingly, these analyses should be interpreted as supplementary and exploratory.
